# A radiomics model predicts the response of patients with advanced gastric cancer to PD-1 inhibitor treatment

**DOI:** 10.18632/aging.203850

**Published:** 2022-01-24

**Authors:** Zhiwen Liang, Ai Huang, Linfang Wang, Jianping Bi, Bohua Kuang, Yong Xiao, Dandan Yu, Ma Hong, Tao Zhang

**Affiliations:** 1 Cancer Center, Union Hospital, Tongji Medical College, Huazhong University of Science and Technology, Wuhan, China; 2 Department of Gastrointestinal Surgery, Union Hospital, Tongji Medical College, Huazhong University of Science and Technology, Wuhan, China; 3 Department of Radiation Oncology, Hubei Cancer Hospital, Tongji Medical College, Huazhong University of Science and Technology, Wuhan, China

**Keywords:** gastric cancer, programmed cell death 1 inhitors, CT image, radiomics, immunotherapy

## Abstract

Programmed cell death 1 (PD1) inhibitors have shown promising treatment effects in advanced gastric cancer, the beneficiary population not definite. This study aimed to construct an individualized radiomics model to predict the treatment benefits of PD-1 inhibitors in gastric cancer. Patients with advanced gastric cancer treated with PD-1 inhibitors were randomly divided into a training set (n = 58) and a validation set (n = 29). CT imaging data were extracted from medical records, and an individual radiomics nomogram was generated based on the imaging features and clinicopathological risk factors. Discrimination performance was evaluated by Harrell’s c-index and receiver operator characteristic (ROC) curve analyses. The areas under the ROC curves (AUCs) were analyzed to predict anti-PD-1 efficacy and survival. We found that the radiomics nomogram could predict the response of gastric cancer to anti-PD-1 treatment. The AUC was 0.865 with a 95% CI of 0.812-0.828 in the training set, while the AUC was 0.778 with a 95% CI of 0.732–0.776 in the validation set. The diagnostic performance of the radiomics was significantly higher than that of the clinical factors (*p* < 0.01). Patients with a low risk of disease progression discriminated by the radiomics nomogram had longer progression-free survival than those with a high risk (6.5 vs. 3.2 months, HR 1.99, 95% CI: 1.19-3.31, *p* = 0.009). The radiomics nomogram based on CT imaging features and clinical risk factors could predict the treatment benefits of PD-1 inhibitors in advanced gastric cancer, enabling it to guide decision-making regarding clinical treatment.

## INTRODUCTION

Gastric cancer is the third most common life-threatening malignancy. Most patients present with progressive disease or metastasis at the time of initial diagnosis [1]. The molecular and biological features of gastric cancers vary from one genetic subtype to another, thus affecting the sensitivity and response of cancers to conventional chemotherapy and single-molecule targeted therapy [2, 3]. Recent studies on immunotherapy have advanced our traditional concepts and tumor treatment approaches. Immune checkpoint inhibitors (ICIs) targeting the PD1/PDL1 axis have shown breakthrough efficacy in a variety of solid tumors, including advanced gastric cancer resistant to chemotherapy after multi-course treatment [3, 4]. Since 2017, anti-PD1 monoclonal antibody has been approved for second-line treatment of advanced gastric cancer. Now, anti-PD1 agents has been approved for first-line treatment of advanced gastric cancer in the whole population [5].

Although PD-1 blockers have revealed inspiring effectiveness in the drug-resistant advanced gastric cancer, it is not well-known who may benefit from the immunotherapy. Accumulated evidence has shown a correlation between the microsatellite instability/mismatch repair (MSI/MMR) status of tumors and the treatment efficacy of ICIs, and MSI-high (MSI-H) or MMR deficiency (dMMR) patients could achieve significant survival benefits from immunotherapy [6, 7], however, this subgroup of patients only accounts for 5–10% of advanced gastric cancer [3, 8]. Other predictive markers such as the programmed death-ligand 1 (PD-L1) expression level, the tumor mutational burden (TMB), and the Epstein–Barr virus (EBV) infection status could be used to guide clinical application, the ability of these factors to predict the treatment outcomes of immunotherapy is controversial due to lack of an optimal efficacy-related cutoff value or low detection rates [3, 4]. In addition to molecular and pathological predictors, some clinical factors such as performance status (PS) and tumor metastasis site could be applied to predict the treatment outcomes of PD-1 inhibitors [9]. Nevertheless, findings from this retrospective study with small sample size are need to be validated in a reproducible clinical trial study.

Currently, researchers are searching for effective and convenient methods to determine tumor heterogeneity and predict treatment outcomes. Computed tomography (CT) is an optimal noninvasive modality for abdominal imaging studies. Nonetheless, conventional CT is not suitable for the quantitative analysis of genetic features or heterogeneity of the tumor or predicting treatment outcomes. Recently, radiomics analysis, a novel method, has been introduced into the field of tumor imaging. It extracts a large number of imaging features, enabling noninvasive quantitative analysis to determine tumor heterogeneity, gene expression profiles, and prediction of treatment outcomes and disease prognosis. As a result, radiomics analysis is also known as an “imaging biomarker” [10, 11]. Several CT-based radiomics studies have shown that radiomics analysis can be used to diagnose different tumors, predict prognoses, assess a specific genetic status, and predict treatment outcomes [12–14]. Hitherto, none of the studies have been conducted to predict the treatment outcomes of immunotherapy for advanced gastric cancer.

Here, we performed a retrospective study in patients with advanced gastric cancer and treated with anti-PD-1 inhibitors. We integrated major clinicopathological factors and imaging features extracted from the previous CT images and generated an innovative individualized radiomics model, in order to characterize responders and non-responder to immunotherapy and determine the clinical application of the radiomics model in patients with advanced gastric cancer.

## MATERIALS AND METHODS

### Study design, patient selection and clinical data collection

This clinical trial is a retrospective observational study that was approved by the Ethics Committee of Huazhong University of Science and Technology, and the requirement for written consent was waived in this study (number UHCT-IEC-SOP-016-02-03).

Patients with advanced gastric cancer receiving PD-1 inhibitors from December 2018 to February 2021 were enrolled. The inclusion criteria were as follows: 1) Patient age was > 18 years and < 75 years old. 2) Relapsed or metastatic inoperable gastric adenocarcinoma was cytologically or pathologically confirmed. 3) Gastric cancer progressed after the first line of standard chemotherapy or a targeted molecular therapy and a subsequent anti-PD-1 therapy for at least 4 cycles. 4) The cancer had at least one evaluable lesion that met the Response Evaluation Criteria in Solid Tumors (RECIST 1.1). 5) CT imaging data before and after anti-PD-1 treatments were complete. 6) Follow-up data were complete. Patients whose gastric lesions were not clearly delineated under CT images or whose metastatic lesions could not be measured or evaluated were excluded from this study. Patients whose survival analyses could not be performed were also excluded from the study. All enrolled cases were further distinguished into a training set and validation set according to the enrolled time stage [15]. The cases enrolled before September 2020 were divided into a training set, while enrolled cases from October 2020 to February 2021 were divided into a validation set.

Clinical features such as age, sex, physical status, tumor differentiation, primary tumor site, and treatment outcomes were retrieved from medical records. Molecular biomarkers including PD-L1, MMR, and HER-2 expression levels were detected by immunohistochemistry and *in situ* hybridization and collected. Per the combined positive score (CPS) of PD-L1, the positive expression of PD-L1 in cancer cells was defined as CPS > 1, and the expression of other molecular markers was interpreted as previously recommended [3].

### Treatment protocol and study endpoints

All patients enrolled in this study had been treated with anti-PD-1 treatment. The outcomes were classified as complete remission (CR), partial remission (PR), stable disease (SD), or progressive disease (PD) per the immune-related RECIST (irRECIST) [16]. Based on the immunotherapy evaluation, SD was also considered to indicate lack of an effect. Therefore, patients with CR, PR, or SD were considered non-PD responders. All patients, including PD and non-PD patients, were followed up for progression-free survival (PFS) and overall survival (OS). PFS was defined as the time from study entry to disease progression or death, and OS was defined as the time from study entry to death or the last follow-up. The primary endpoints were the radiomics prediction accuracy and effectiveness of cancer response to PD-1 inhibitor treatment. The secondary endpoint was survival prediction, including PFS and OS, by the radiomics model.

### CT imaging data collection

All patients in this study were subjected to contrast-enhanced CT scans (16-MDCT, Brilliance Big bore Philips Health care, Cleveland, OH, USA) at 120 kV. The slice intervals were 5 mm. Portal venous phases of enhanced abdominal CT images were reviewed to determine the tumor location, size, shape, internal echoes of lymph nodes, and the spatial correlation between the tumor and surrounding normal tissues.

### Tumor area segmentation and radiomic feature extraction

The primary tumor area on each slice of transverse CT images was manually segmented and set as the region of interest (ROI) by two independent experienced radiologists who were blinded to this study design though the commercial software AccuContuor (Manteia Medical Technologies Co, XiaMen, China). Both radiologists repeated the segmentation procedure twice. The ROI segmented images were resampled with an isotropic voxel size (2×2×2 mm^3^) and interpolated with the Bspline algorithm. Filters including wavelet and Laplacian of Gaussian (LOG) with different sigma values (0.5–5 with steps 0.5) were applied to all the segmented images. Subsequently, the images were discretized into fixed bin widths. A total of 103 radiomics features were extracted from the original segmented images including 13 shape-based features,17 first-order based features, 22 texture features consisting of gray level cooccurrence matrix (GLCM) features, 16 gray level run length matrix (GLRLM) features, 16 gray level size zone matrix (GLSZM) features, 14 gray level dependence matrix (GLDM) features, and 5 neighboring gray-tone difference matrix (NGTDM) features, through the ontology-guided radiomics analysis workflow (O-RAW) package [17]. For filtered images, the same radiomics features were extracted except the shape-based features. Therefore, a total of 1723 radiomics features were extracted and analyzed in each patient.

### Feature selection and radiomics score calculation

All extracted radiomic features were normalized with z scores. Intra- and interobserver reproducibility were evaluated by intraclass correlation coefficients (ICCs), and features with ICCs less than 0.75 were excluded. To avoid collinearity, redundant features (correlation coefficients > 0.9) were excluded by Pearson’s correlation analysis, and zero importance features were removed by a gradient boosting method (LightGBM) using the feature-selector python package (https://github.com/WillKoehrsen/feature-selector). An all-relevant feature selection method was applied to determine the final significant features [18]. A support vector machine (SVM) model and logistic regression model were constructed and compared using 5-fold cross validation and 10 repeats [19]. A radiomics score (rad-score) was built based on the linear combination of the features weighted by the selected model.

### Nomogram model building and verification

In order to determine the contributing factors to immunotherapy response, Multivariate logistic regression was used to analyze the relationship of the clinical risk factors including age, sex, primary tumor site, and molecular biomarkers as PD-L1, MMR, HER-2 expression levels, etc., as well as the radiomics score with the immunotherapy response. Radiomics and clinical models were established based on the high-risk clinical factors combined with or without the rad-score, respectively. The discrimination performance of the two models was evaluated by Harrell’s c-index and receiver operator characteristic (ROC) curves in both the training and validation sets. The ROC curves between the two models were compared by the Delong test. The calibration curve and Hosmer–Lemeshow test were used to identify the predictive accuracy of the radiomics model in both sets.

### Clinical application evaluation

A decision curve analysis was performed to compare the net benefit difference of the two models and determine the corresponding clinical application value. Based on the total points of the radiomics nomogram, a high or a low-risk probability of disease progression after PD-1 inhibitor treatment was determined. Finally, the impact on survival, such as PFS and OS, was analyzed through survival analysis.

### Statistical analysis

Statistical analyses were performed with SPSS (version 26, Chicago, IL, USA) and R software (version 3.6.2, http://www.Rproject.org). Categorical variables were analyzed using the chi-square test or Fisher’s exact test, and continuous variables were analyzed using Student’s t test or Mann–Whitney U test. Correlation analysis was assessed by the Pearson correlation test. Nomograms and calibration curves were plotted by the R package ‘RMS’. The decision curves were plotted by the R package ‘RMDA’. All of the R packages can be installed directly in the R console by commands. The ROC curves were plotted by the python package ‘Scikit-learn’ (https://github.com/scikit-learn/scikit-learn). The Kaplan–Meier method was used for survival analysis, and the log-rank test was used to analyze survival data. Cox regression analysis was performed to identify survival-related factors. For all tests, two-sided *p*< 0.05 was considered statistically significant.

## RESULTS

### Baseline features

**Figure 1:**
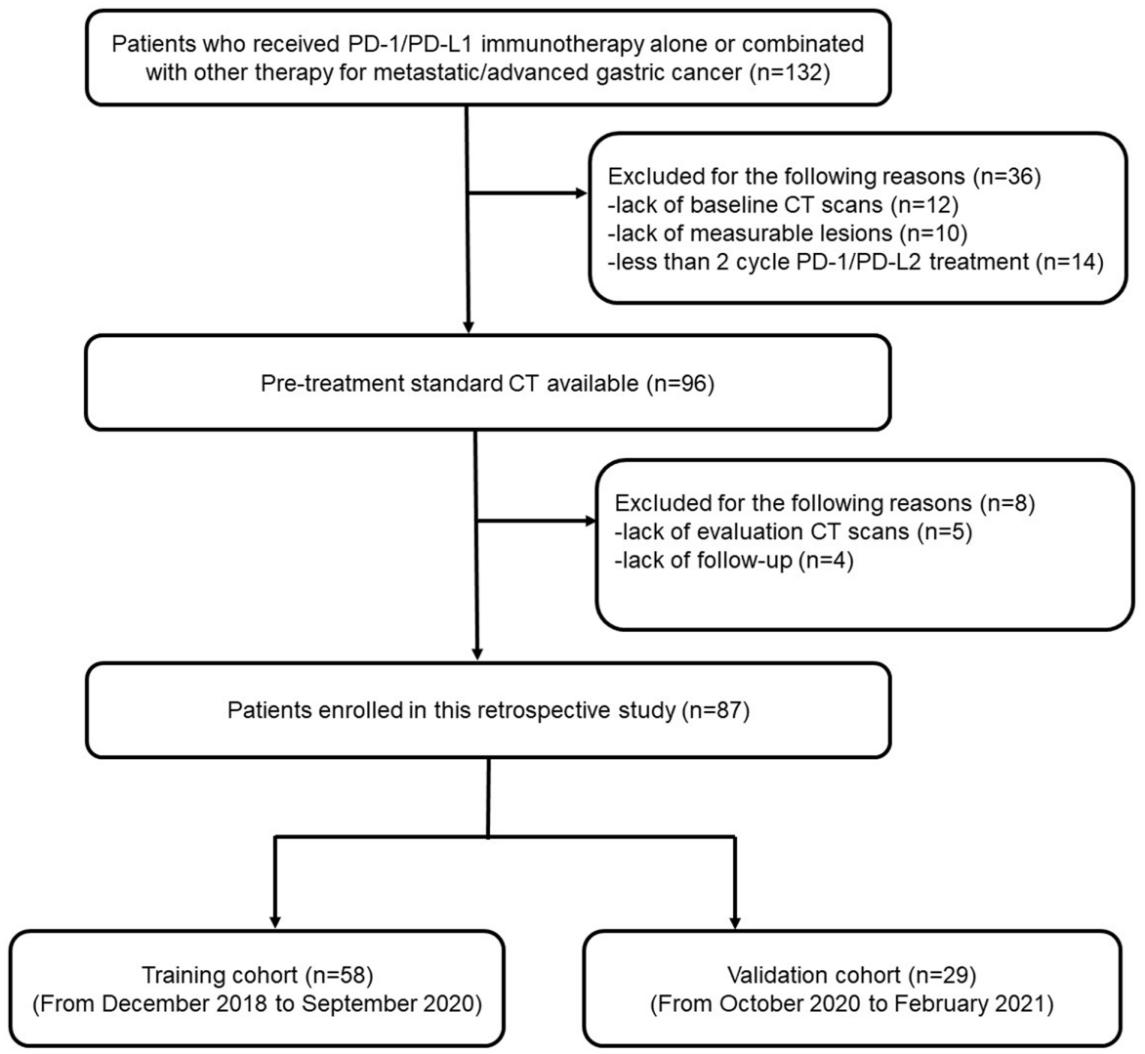
A flow chart of patient enrollment.

A total of 87 patients with advanced gastric cancer were included in this study. All enrolled patients were treated with PD-1 inhibitors (toripalimab) after first-line standard chemotherapy, and enrolled patients were divided into a training set (n = 58) and a validation set (n = 29) (Figure 1). Every patient in the cohort was classified as a PD responder or non-PD responder according to the treatment efficacy. The baseline features are listed in Table 1. The median age of the participants was 55 (range: 28–76) years old, and 59.7% of the patients were males. No significant difference was observed in any of these factors, including age, sex, primary tumor site, tumor differentiation, and genetic status (MMR, PD-L1), between the two responders in either the training or validation cohort. However, a significant difference was observed in the level of serum carcinoembryonic antigen (CEA) and the metastatic tumor site between the responder and non-responder groups. Patients with elevated CEA levels exhibited a significantly higher response rate to PD-1 inhibitors than those with normal CEA levels before treatment. In addition, the response rate to PD-1 inhibitors was significantly higher in patients without peritoneal metastasis (Table 1).

**Table 1:** Baseline characteristics of enrolled advanced gastric cancer patients in the training group and validation group.

**Clinical characteristics**	**Training cohort (n= 58)**			**Validation cohort (n= 29)**		
	**Non-PD responder (n=30, %)**	**PD responder (n=28, %)**	**P value**	**Non-PD responder (n=15, %)**	**PD responder (n=14, %)**	**P value**
**Age**			0.380			0.315
≤60y	17 (56.7)	19 (67.9)		8 (53.3)	10 (71.4)	
>60y	13 (43.3)	9 (32.1)		7 (46.7)	4 (28.6)	
**Gender**			0.457			0.572
Male	21 (70.0)	17 (60.7)		8 (53.3)	6 (42.9)	
female	9 (30.0)	11 (39.3)		7 (46.7)	8 (57.1)	
**Tumor differentiation**			0.825			0.280
Well or moderate	12 (40.0)	12 (42.9)		6 (40.0)	3 (21.4)	
Poor	18 (60.0)	16 (57.1)		9 (60.0)	11 (78.6)	
**Primary tumor location**						
Cardia	4 (13.3)	7 (25.0)	0.257	1 (6.7)	2 (14.3)	0.464
Body	7 (23.3)	9 (32.1)	0.453	3 (20.0)	3 (21.4)	0.924
Antrum	9 (30.0)	7 (25.0)	0.670	5 (33.3)	3 (21.4)	0.543
Whole	10 (33.3)	5 (17.9)	0.178	6 (40.0)	6 (42.9)	0.765
**Metastasis status**			0.002*			0.017*
Peritoneal metastasis	13(43.3)	23(82.1)		10 (66.7)	14 (100.0)	
Non-Peritoneal metastasis	17(56.7)	5(17.9)		5 (33.3)	0 (0.0)	
**Pretreatment CEA level (ng/ml)**			0.003*			0.035*
Elevated	21 (70.0)	9 (32.1)		9 (60.0)	3 (21.4)	
Normal	9 (30.0)	19 (67.9)		6 (40.0)	11 (78.6)	
**Pretreatment CA199 level (ng/ml)**			0.585			0.355
Elevated	15 (50.0)	12 (42.9)		6 (40.0)	8 (57.1)	
Normal	15 (50.0)	16 (57.1)		9 (60.0)	6 (42.9)	
**MMR/MSI status**			0.955			0.707
pMMR/MSS	18 (60.0)	17 (60.7)		11 (68.8)	9 (69.2)	
Not available	12 (40.0)	11 (39.3)		5 (31.2)	4 (30.8)	
**PD-L1 expression**			0.677			0.431
High expression	3 (10.0)	2 (7.2)		8 (53.3)	6 (42.9)	
Low expression	16 (53.3)	16 (57.1)		5 (33.3)	7 (50.0)	
Not available	11 (36.7)	10 (35.7)		2 (13.4)	1 (7.1)	
**Her-2 Expression**			0.142			0.236
0	14 (46.7)	2 (7.2)		6 (40.0)	4 (28.6)	
1-2	10 (33.3)	16 (57.1)		1 (6.7)	3 (21.4)	
Not available	6 (20.0)	10 (35.7)		8 (53.3)	7 (50.0)	
**anti-PD1 treatment**			0.191			0.949
With chemotherapy	28 (93.3)	23 (82.1)		14 (93.3)	12 (85.7)	
Without chemotherapy	2 (6.7)	5 (17.9)		1 (6.7)	2 (14.3)	

Abbreviation: CEA, carcinoembryonic antigen; CA199, carbohydrate antigen 19-9; MSI / MMR, microsatellite instability / mismatch repair; pMMR, MMR proficient; HER-2, human epidermal growth factor receptor 2; PD1, programmed cell death 1; PD-L1, programmed death-ligand 1.

P value means the difference between responder and non-responder group, *p <0.05 statistically significant.

### Radiomics feature selection

**Figure 2:**
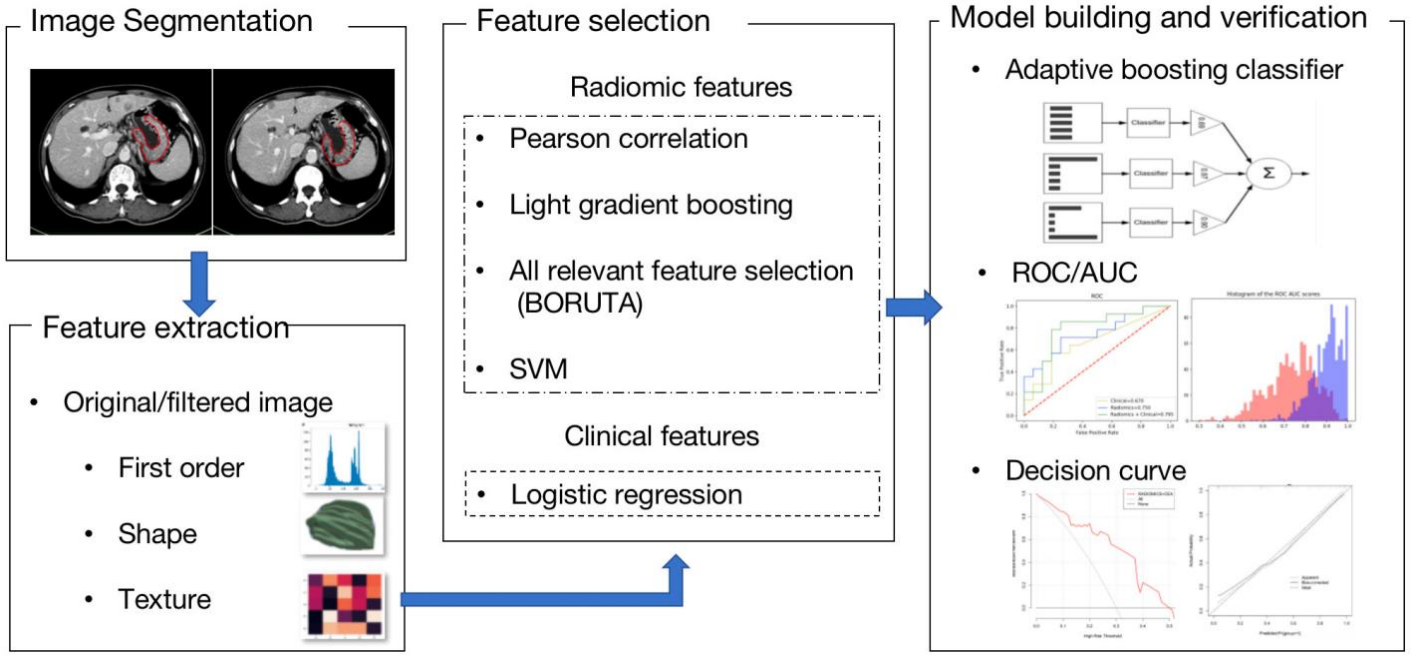
**A flow chart of the radiomics analysis.** Based on CT images, important imaging features were screened and combined with important clinical risk factors to generate radiomics nomograms. The performance and clinical utility of the radiomics model to predict anti-PD-1 treatment efficacy were evaluated through receiver operator characteristic (ROC), calibration and decision curve analyses. Survival prediction was also explored.

**Figure 3:**
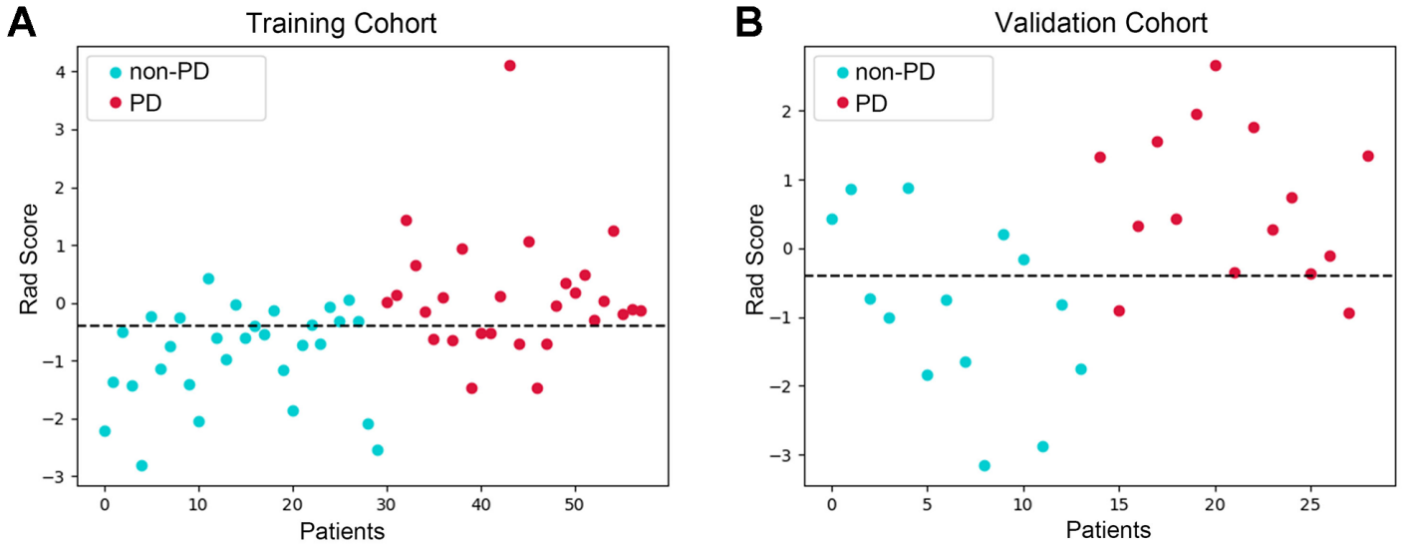
**Differences in rad-scores between anti-PD1 treatment responders were detected.** Scatter plot of the rad-score in the training set (**A**) and in the validation set (**B**). The blue dots represent the rad-score of patients with PD, and red dots represent the rad-score of patients without PD. Our analyses revealed that the patients with PD after immunotherapy had obviously higher rad-scores than those without PD in the training and validation set.

The radiomics feature selection and relevant workflow are illustrated in Figure 2. The primary tumor area was manually segmented and set as the ROI. After extracting important radiomics features and excluding the features with ICCs less than 0.75, 1244 radiomics features were used for further analyses. Colinear feature analysis resulted in 329 features being relevant, and final zero-importance feature analysis revealed that 18 features were important. Of them, 7 features showed significant differences between the response and nonresponse groups. Among these 7 radiomics features, 3 were identified as most relevant at the end of selections. Logistic regression and SVM models were built based on the 3 selected radiomic features to classify the PD responders and non-PD responders. The AUCs of the logistic regression and SVM models were 0.702 (95% CI: 0.694-0.711) and 0.695 (95% CI: 0.685-0.706) in the training set, respectively. More comparison results can be found in Supplementary Table 1. The logistic regression model was chosen for calculating rad-scores for each patient, and the rad-score formulas are listed in the Supplementary Text. Our analyses found that the patients with PD after immunotherapy had obviously higher rad-scores than those without PD in the training set (0.11 ± 1.10 vs. - 0.90 ± 0.68, *p* < 0.001) and in the validation set (0.64 ± 1.18 vs.-0.88 ± 1.62, *p* = 0.001) (Figure 3A, 3B).

### The radiomics model predicts immunotherapy efficacy better than the clinical model

**Figure 4:**
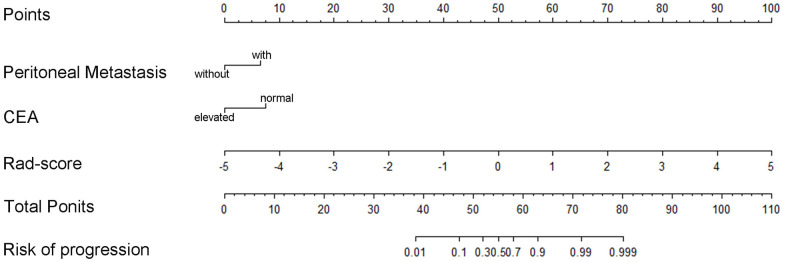
The radiomics nomogram for predicting anti-PD-1 efficacy in patients with advanced gastric cancer based on the rad-score with clinicopathological risk factors for CEA level and tumor metastasis site.

**Figure 5:**
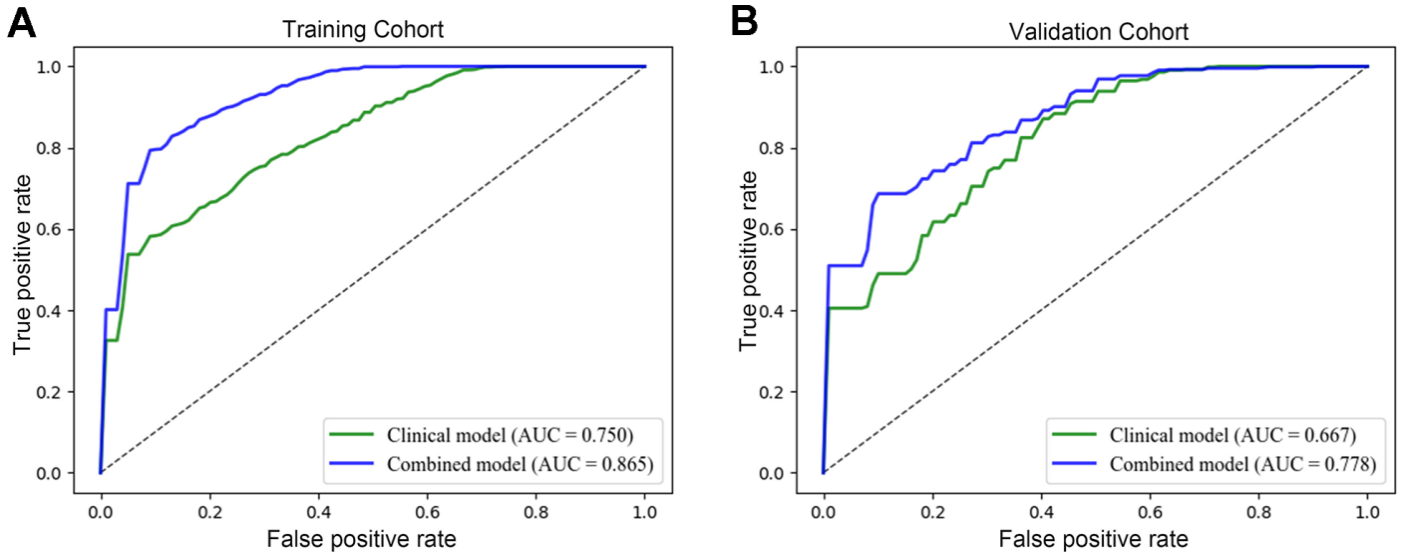
**Evaluation of the performance of the radiomics model.** The operating characteristic curve analysis of the clinical and radiomics models was plotted in the training set (**A**) and in the validation set (**B**). The AUCs of the radiomics model were 0.865 (95% CI: 0.812-0.828) and 0.778 (95% CI: 0.732–0.776) in the training and validation datasets. While the AUCs of the clinical model were 0.750 (95% CI: 0.718–0.734) and 0.667 (95% CI: 0.665–0.724), respectively.

**Figure 6:**
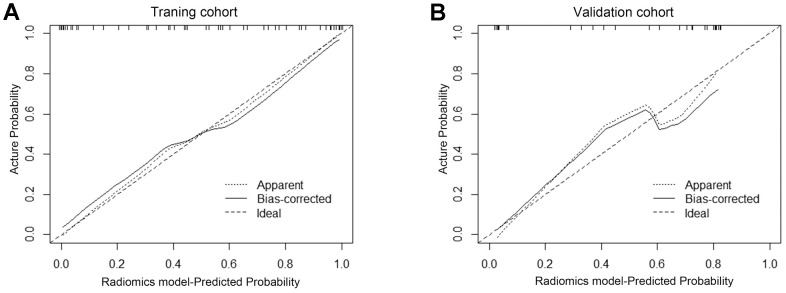
**The calibration curve showed the consistency between the predicted and observed probabilities.** Calibration curves were plotted in the training set (**A**) and in the validation set (**B**). The 45-degree reference line represents an ideal standard calibration line. The solid line represents the prediction performance of the radiomics model without overfitting correction. The dotted line is the performance of the nomogram after bootstrap correction, which is used as the prediction of future accuracy. The closer the prediction curve is to the standard curve, the better the prediction ability of the nomogram.

**Figure 7:**
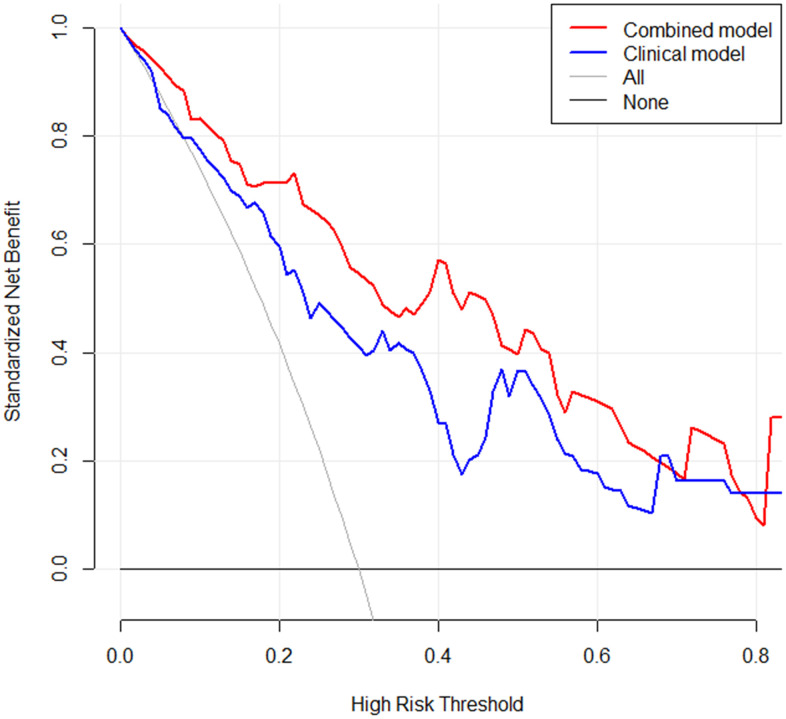
**A decision curve was applied to compare the difference in net benefit of the clinical model and radiomics model in the validation cohort.** X-axis means threshold probability, Y-axis means net benefit. The decision curve showed that when the threshold probability exceeds 10%, and the net benefit of clinical application in radiomics model would exceed the clinical model.

To identify the clinical risk factors attributing to the therapeutic effect, we performed multi-factor regression analysis. The results manifested that the serum CEA level and the sites of metastasis were two major clinical risk factors contributing to the immunotherapy response (Table 2). Thus, we developed a clinical model consists of two major clinical risk factors and a radiomics model that integrated two clinical risk factors and rad-scores (Figure 4). Our study revealed that the AUCs of the radiomics model were 0.865 (95% CI: 0.812-0.828) and 0.778 (95% CI: 0.732–0.776) in the training and validation datasets. While the AUCs of the clinical model were 0.750 (95% CI: 0.718–0.734) and 0.667 (95% CI: 0.665–0.724), respectively (Table 3 and Figure 5A, 5B). The nomogram calibration curve showed good agreement between the predicted and observed outcomes. Compared with the clinical model, the radiomics model not only performed a more accurate diagnosis than the clinical model (*p* < 0.01) (Figure 5A, 5B) but also better predicted probabilities of anti-PD-1 efficacy that were consistent with the actual probability in both the training and validation cohorts (Figure 6A, 6B). The decision curve showed that the radiomics model has a better net benefit rate than the clinical model in further (Figure 7).

**Table 2:** Risk factors for anti-PD1 response in advanced gastric cancer.

**Variable**	**Clinical model**			**Radiomics model**		
	**HR**	**95%CI**	**P value**	**HR**	**95%CI**	**P value**
CEA level	0.268	0.103-0.696	0.007*	0.120	0.033-0.439	0.001*
Peritoneal metastasis	5.787	1.931-17.343	0.002*	4.628	1.225-17.489	0.024*
Rad-score	NA	NA	NA	6.167	2.356-16.144	<0.001*

Abbreviation: programmed cell death 1, PD1; CEA, carcinoembryonic antigen; HR, Hazard Ratio, 95%CI, 95% confidence intervals; NA, not available.

P value means the difference between clinical and radiomics model, *p <0.05 statistically significant.

**Table 3:** Performance evaluation of the clinical and radiomic model.

**Index**	**Training cohort**		**Validation cohort**	
	**Clinical model**	**Radiomic model**	**Clinical model**	**Radiomic model**
Sensitivity	0.752	0.836	0.738	0.775
Specificity	0.696	0.801	0.674	0.742
AUC	0.750	0.865*	0.667	0.778*
95%CI	0.718-0.734	0.812-0.828	0.665-0.724	0.732-0.776

Abbreviation: AUC, area under curve; CI, confidence interval.

*means statistically significant difference existed between clinical model and radiomic model in AUC value (*p* < 0.05).

### The radiomics model accurately predicts the progression-free survival

**Figure 8:**
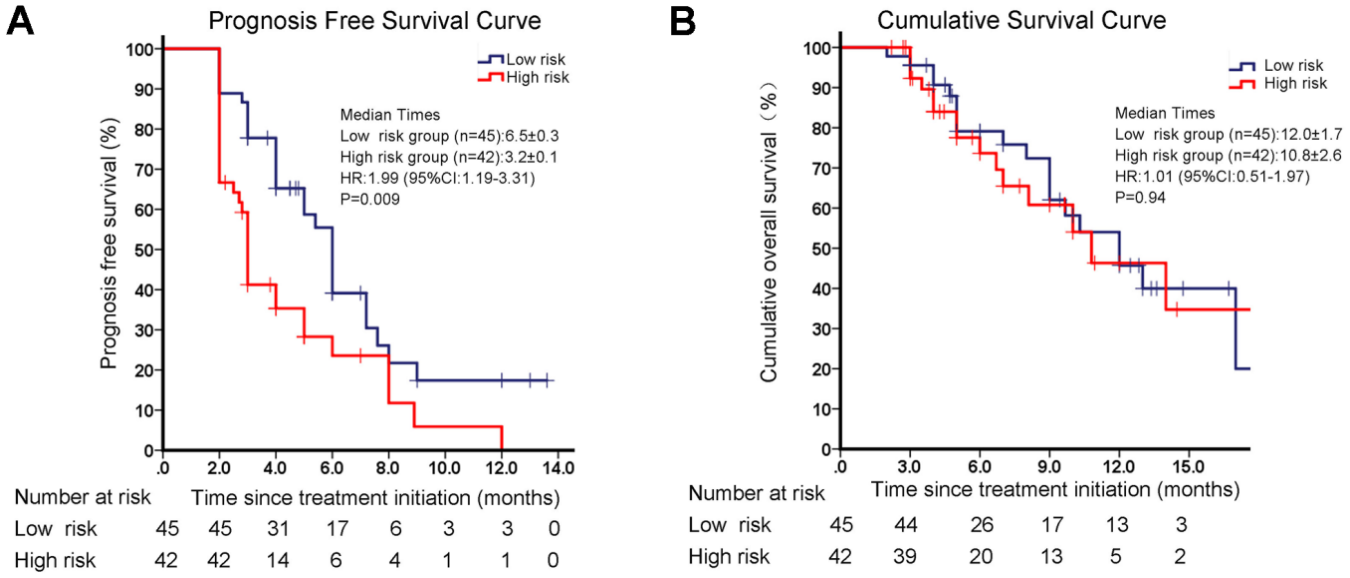
**Survival analyses in different groups of disease progression risk classified by the radiomics model.** (**A**) Progression-free survival (PFS) in different groups of disease progression risk. (**B**) Overall survival (OS) in different groups of disease progression risk.

All enrolled patients were followed up to monitor PFS and OS. Two patients’ follow up data were lost, and 85 patients had completed survival data. Among them, 61 patients had PD, and 35 patients died. The median follow-up time was 10 (range: 3–23) months. Based on the radiomics nomogram, we identified the cutoff value for total points as 54 for classifying patients into high risk of disease progression and low-risk disease progression groups after PD-1 inhibitor treatment. Patients with more than 54 total points were grouped in the high-risk group, and the others were grouped in the low-risk group. We found that there was no difference in OS between the high- and low-risk groups (12.0 vs. 10.8 months, *p* = 0.94). However, the median PFS in the low-risk group was 6.5 months, which was significantly longer than the 3.2 months in the high-risk group (hazard ratio (HR): 1.99, 95% CI: 1.19-3.31, *p* = 0.009) (Figure 8A, 8B). These data indicated that patients with low-risk values discriminated by the radiomics model had better survival outcomes after immunotherapy. Our univariate and multivariate regression analyses confirmed that the risk value determined by the radiomics model well reflected the prognosis of PFS (Table 4).

**Table 4:** Uni- and multivariable cox regression analysis of predictors for progression-free survival.

	**Progression-free survival**					
	**Univariable analysis**			**Multivariable analysis**		
	**HR**	**95%CI**	**P value**	**HR**	**95%CI**	**P value**
Age≤60y vs. >60y	0.731	0.396-1.349	0.316			
Gender male vs. female	1.158	0.397-3.371	0.788			
Tumor DifferentiationWell or moderate vs. poor	0.534	0.245-1.163	0.114			
Tumor locationWhole vs. no-whole	1.523	0.550-4.218	0.419			
cT stage	1.941	0.795-4.741	0.145			
cT1-2 vs. cT3-4						
cN stage	1.066	0.485-2.344	0.874			
N+ vs. N0						
Peritoneal metastasis Yes vs. No	1.726	0.880-3.387	0.112			
Pre-treatment CEA levelNormal vs. Elevated	0.492	0.273-0.886	0.018*	0.755	0.392-1.452	0.400
Pre-treatment CA199 level Normal vs. Elevated	1.388	0.758-2.544	0.288			
Anti-PD1+chemotherapyYes vs. No	0.807	0.111-5.891	0.833			
High vs Low risk value	2.992	1.648-5.435	<0.001*	2.647	1.367-5.128	0.004*

Abbreviation: CEA, carcinoembryonic antigen; CA199, carbohydrate antigen 19-9; HR, Hazard Ratio; 95%CI, 95% confidence intervals. *p < 0.05 statistically significant.

## DISCUSSION

ICIs mainly target a tumor’s immune microenvironment and function by reactivating the host’s immune cells. These biological agents have distinct effects from other conventional antitumor drugs. They were either effective or ineffective at all (all or none) and as such they may substantially benefit some but not all patients [3, 4]. Therefore, it is essential to identify the biomarkers in tumors that can be used to predict the tumor response to immunotherapy and the treatment outcomes of cancer patients. In this study, we developed a noninvasive CT-based radiomics model to predict the outcomes of anti-PD-1 therapy in patients with advanced gastric cancer, distinguish the responders from non-responders to PD-1 inhibitor treatment and predict patient survivals before therapy. Our study found that both serum carcinoembryonic antigen levels and tumor metastasis sites were correlated with the cancer response to PD-1 inhibitor treatment and could be integrated with imaging features to generate a radiomics model, and the radiomics model exhibited a good diagnostic performance and clinical application. Moreover, patients with a low risk of disease progression discriminated by the radiomics nomogram were demonstrated to have a better survival benefit. Thus, our study reveals that the CT-based radiomics nomogram could be applied to predict the response and outcomes to PD-1 inhibitor immunotherapy in patients with advanced gastric cancer. The validated radiomic model could be used to guide the decision-making for gastric cancer treatment.

The MSI/MMR status, PD-L1 expression, tumor mutation burden, and EBV infection are known to be crucial factors contributing to the efficacy of immunosuppressive agents [3, 4]. In this study, we examined the correlation between the molecular status of these clinical factors and the efficacy of PD-1 inhibitor treatment and found that none of the enrolled cases had MSI-H/dMMR status. However, more than 50% of them responded to anti-PD-1 treatment and showed effectiveness. Our study indicates that the incidence of MSI-H tumors is low in patients with advanced gastric cancer in our community. In agreement with the findings from other studies, we observed that some of patients with MSI-low (MSI-L)/MMR proficiency (pMMR) still had beneficial responses to PD-1 inhibitor treatment [20, 21]. We did not detect a significant correlation between the anti-PD-1 treatment response and any factors of the Her-2/Neu expression level, or the PD-L1 expression level, indicating that information provided from current molecular detections is not sufficient to predict the efficacy of immunotherapy.

In this study, we found that pretreatment serum CEA levels and tumor metastasis status were two major predictors of anti-PD-1 treatment outcomes. Serum CEA has been the most common tumor marker of gastrointestinal tumors, and an elevated level of serum CEA indicates the presence of tumor progression and relapse [22, 23]. A change in the CEA level after treatment is an indicator of treatment response, although the accuracy and clinical relevance are low [23, 24]. Consistent with previous reports, we demonstrated that patients with elevated CEA levels were more likely to benefit from anti-PD-1 treatment than those with normal CEA levels before treatment. In addition, tumor metastasis status has also been regarded as a vital factor in the efficacy of anti-PD-1 therapy [8]. Our study showed that patients without peritoneal metastasis were more likely to respond to immunotherapy than those with peritoneal metastasis. When we combined the CEA level with peritoneal metastasis status and generated a clinical model, we could increase the prediction accuracy. Above data indicated that clinical factors associated with tumoral heterogeneity and burden could be integrated into our radiomics model to predict the immunotherapy response.

The antitumor mechanism of immunotherapy differs from that of other drugs, and some specific imaging responses might be observed after immunotherapy, such as persistent SD, pseudoprogression, or superprogression [25], thus complicating the interpretation of imaging data and clinical decision-making. As a novel diagnostic tool, radiomics could identify tumor heterogeneity and predict antitumor efficacy through screening and quantitative analysis of key imaging features. Gastric cancer has been characterized by high genetic and immunological heterogeneity [26], and tumor feature identification in a noninvasive and convenient manner pretreatment would help clinicians develop an efficient treatment plan. Wang et al. conducted a retrospective analysis and found that preoperative CT-based radiomics analysis is an effective tool for screening progressive gastric cancer and deducing the status of human epidermal growth factor receptor 2 (HER-2) [12, 13]. Huang et al. and Sun et al. successfully applied radiomics analysis before surgery to predict the peritoneal metastasis of gastric cancer and the response to neoadjuvant therapy [27, 28]. However, studies on the prediction and analysis of the treatment outcomes of immunotherapy for gastric cancer are lacking. Here, we demonstrated that radiomics features based on pretreatment CT image could effectively predict the efficiency of anti-PD1 treatment when these radiomics features were combined with CEA level and tumor metastasis status to construct a radiomics model. The validated radiomics model possesses superior discrimination power to the model generated from clinical factors alone.

There are a few limitations in our study. First, this is a single-center retrospective study with a limited sample size. The sample size could have been larger if multiple centers were involved in the study and more patients with advanced gastric cancer had participated in the screening. Second, because the imaging features were extracted from a sample of limited size and might not be stable, there is still room for improvement to increase the prediction efficiency (e.g., AUC) and accuracy of the radiomics model with a larger sample size. Third, PD-1 inhibitors (toripalimab) were selected for analysis in the study, although they are not an indication for gastric cancer. Existing clinical studies have confirmed its applicability in advanced gastric cancer [29, 30]. In our research, we fixed the type of PD1 inhibitor to reduce the interference of confounding factors and obtained ethical support. Finally, we did not screen out the molecular markers related to efficacy because MSI-H was not found in the enrolled patients, and only 21.8% of enrolled patients showed a high level of PD-L1 expression. The molecular markers identified as related to the efficacy of immunotherapy have a low positive clinical probability and limited predictive ability. Therefore, a multicenter prospective study with a large sample size is needed to validate our findings.

In summary, we developed and validated a CT-based radiomics model that could be used to predict the treatment benefits of PD-1 inhibitors in patients with advanced gastric cancer. Our study indicates that the radiomics model is a promising tool for prognosis and clinical decision-making.

## Supplementary Material

Supplementary Text

Supplementary Table 1

## References

[1] Bray F, Ferlay J, Soerjomataram I, Siegel RL, Torre LA, Jemal A. Global cancer statistics 2018: GLOBOCAN estimates of incidence and mortality worldwide for 36 cancers in 185 countries. CA Cancer J Clin. 2018; 68:394–424. 10.3322/caac.21492 30207593

[2] Cancer Genome Atlas Research Network. Comprehensive molecular characterization of gastric adenocarcinoma. Nature. 2014; 513:202–9. 10.1038/nature13480 25079317 PMC4170219

[3] Zhang Z, Xie T, Zhang X, Qi C, Shen L, Peng Z. Immune checkpoint inhibitors for treatment of advanced gastric or gastroesophageal junction cancer: Current evidence and future perspectives. Chin J Cancer Res. 2020; 32:287–302. 10.21147/j.issn.1000-9604.2020.03.02 32694895 PMC7369180

[4] Bai R, Chen N, Liang T, Li L, Lv Z, Lv X, Cui J. Novel Frontiers of Treatment for Advanced Gastric or Gastroesophageal Junction Cancer (GC/GEJC): Will Immunotherapy Be a Future Direction? Front Oncol. 2020; 10:912. 10.3389/fonc.2020.00912 32793461 PMC7386300

[5] Eso Y, Shimizu T, Takeda H, Takai A, Marusawa H. Microsatellite instability and immune checkpoint inhibitors: toward precision medicine against gastrointestinal and hepatobiliary cancers. J Gastroenterol. 2020; 55:15–26. 10.1007/s00535-019-01620-7 31494725 PMC6942585

[6] Zhao P, Li L, Jiang X, Li Q. Mismatch repair deficiency/microsatellite instability-high as a predictor for anti-PD-1/PD-L1 immunotherapy efficacy. J Hematol Oncol. 2019; 12:54. 10.1186/s13045-019-0738-1 31151482 PMC6544911

[7] Polom K, Marano L, Marrelli D, De Luca R, Roviello G, Savelli V, Tan P, Roviello F. Meta-analysis of microsatellite instability in relation to clinicopathological characteristics and overall survival in gastric cancer. Br J Surg. 2018; 105:159–67. 10.1002/bjs.10663 29091259

[8] Mishima S, Kawazoe A, Nakamura Y, Sasaki A, Kotani D, Kuboki Y, Bando H, Kojima T, Doi T, Ohtsu A, Yoshino T, Kuwata T, Tsuji A, Shitara K. Clinicopathological and molecular features of responders to nivolumab for patients with advanced gastric cancer. J Immunother Cancer. 2019; 7:24. 10.1186/s40425-019-0514-3 30704511 PMC6357506

[9] Janjigian YY, Shitara K, Moehler M, Garrido M, Salman P, Shen L, Wyrwicz L, Yamaguchi K, Skoczylas T, Campos Bragagnoli A, Liu T, Schenker M, Yanez P, et al. First-line nivolumab plus chemotherapy versus chemotherapy alone for advanced gastric, gastro-oesophageal junction, and oesophageal adenocarcinoma (CheckMate 649): a randomised, open-label, phase 3 trial. Lancet. 2021; 398:27–40. 10.1016/S0140-6736(21)00797-2 34102137 PMC8436782

[10] Liu Z, Wang S, Dong D, Wei J, Fang C, Zhou X, Sun K, Li L, Li B, Wang M, Tian J. The Applications of Radiomics in Precision Diagnosis and Treatment of Oncology: Opportunities and Challenges. Theranostics. 2019; 9:1303–22. 10.7150/thno.30309 30867832 PMC6401507

[11] Limkin EJ, Sun R, Dercle L, Zacharaki EI, Robert C, Reuzé S, Schernberg A, Paragios N, Deutsch E, Ferté C. Promises and challenges for the implementation of computational medical imaging (radiomics) in oncology. Ann Oncol. 2017; 28:1191–206. 10.1093/annonc/mdx034 28168275

[12] Wang Y, Liu W, Yu Y, Han W, Liu JJ, Xue HD, Lei J, Jin ZY, Yu JC. Potential value of CT radiomics in the distinction of intestinal-type gastric adenocarcinomas. Eur Radiol. 2020; 30:2934–44. 10.1007/s00330-019-06629-3 32020404

[13] Wang Y, Yu Y, Han W, Zhang YJ, Jiang L, Xue HD, Lei J, Jin ZY, Yu JC. CT Radiomics for Distinction of Human Epidermal Growth Factor Receptor 2 Negative Gastric Cancer. Acad Radiol. 2021; 28:e86–92. 10.1016/j.acra.2020.02.018 32303442

[14] Liu Z, Zhang XY, Shi YJ, Wang L, Zhu HT, Tang Z, Wang S, Li XT, Tian J, Sun YS. Radiomics Analysis for Evaluation of Pathological Complete Response to Neoadjuvant Chemoradiotherapy in Locally Advanced Rectal Cancer. Clin Cancer Res. 2017; 23:7253–62. 10.1158/1078-0432.CCR-17-1038 28939744

[15] Park KJ, Lee JL, Yoon SK, Heo C, Park BW, Kim JK. Radiomics-based prediction model for outcomes of PD-1/PD-L1 immunotherapy in metastatic urothelial carcinoma. Eur Radiol. 2020; 30:5392–403. 10.1007/s00330-020-06847-0 32394281

[16] Seymour L, Bogaerts J, Perrone A, Ford R, Schwartz LH, Mandrekar S, Lin NU, Litière S, Dancey J, Chen A, Hodi FS, Therasse P, Hoekstra OS, et al, and RECIST working group. iRECIST: guidelines for response criteria for use in trials testing immunotherapeutics. Lancet Oncol. 2017; 18:e143–52. 10.1016/S1470-2045(17)30074-8 28271869 PMC5648544

[17] Shi Z, Traverso A, van Soest J, Dekker A, Wee L. Technical Note: Ontology-guided radiomics analysis workflow (O-RAW). Med Phys. 2019; 46:5677–84. 10.1002/mp.13844 31580484 PMC6916323

[18] Shi L, Westerhuis JA, Rosén J, Landberg R, Brunius C. Variable selection and validation in multivariate modelling. Bioinformatics. 2019; 35:972–80. 10.1093/bioinformatics/bty710 30165467 PMC6419897

[19] Fan Y, Jiang S, Hua M, Feng S, Feng M, Wang R. Machine Learning-Based Radiomics Predicts Radiotherapeutic Response in Patients With Acromegaly. Front Endocrinol (Lausanne). 2019; 10:588. 10.3389/fendo.2019.00588 31507537 PMC6718446

[20] Fukuoka S, Hara H, Takahashi N, Kojima T, Kawazoe A, Asayama M, Yoshii T, Kotani D, Tamura H, Mikamoto Y, Hirano N, Wakabayashi M, Nomura S, et al. Regorafenib Plus Nivolumab in Patients With Advanced Gastric or Colorectal Cancer: An Open-Label, Dose-Escalation, and Dose-Expansion Phase Ib Trial (REGONIVO, EPOC1603). J Clin Oncol. 2020; 38:2053–61. 10.1200/JCO.19.03296 32343640

[21] Janjigian YY, Bendell J, Calvo E, Kim JW, Ascierto PA, Sharma P, Ott PA, Peltola K, Jaeger D, Evans J, de Braud F, Chau I, Harbison CT, et al. CheckMate-032 Study: Efficacy and Safety of Nivolumab and Nivolumab Plus Ipilimumab in Patients With Metastatic Esophagogastric Cancer. J Clin Oncol. 2018; 36:2836–44. 10.1200/JCO.2017.76.6212 30110194 PMC6161834

[22] Nakanishi K, Kanda M, Umeda S, Tanaka C, Kobayashi D, Hayashi M, Yamada S, Kodera Y. The levels of SYT13 and CEA mRNAs in peritoneal lavages predict the peritoneal recurrence of gastric cancer. Gastric Cancer. 2019; 22:1143–52. 10.1007/s10120-019-00967-3 31055693

[23] Zhou Q, Lan X, Li N, Yuan D, Zhang J. Analysis of Prognostic Factors and Design of Prognosis Model for Patients with Stage IV Gastric Cancer Following First-Line Palliative Chemotherapy. Cancer Manag Res. 2020; 12:10461–8. 10.2147/CMAR.S263320 33122945 PMC7588669

[24] Abbas M, Ahmed A, Khan GJ, Baig MM, Naveed M, Mikrani R, Cao T, Naeem S, Shi M, Dingding C. Clinical evaluation of carcinoembryonic and carbohydrate antigens as cancer biomarkers to monitor palliative chemotherapy in advanced stage gastric cancer. Curr Probl Cancer. 2019; 43:5–17. 10.1016/j.currproblcancer.2018.08.003 30172422

[25] Borcoman E, Kanjanapan Y, Champiat S, Kato S, Servois V, Kurzrock R, Goel S, Bedard P, Le Tourneau C. Novel patterns of response under immunotherapy. Ann Oncol. 2019; 30:385–96. 10.1093/annonc/mdz003 30657859

[26] Derks S, de Klerk LK, Xu X, Fleitas T, Liu KX, Liu Y, Dietlein F, Margolis C, Chiaravalli AM, Da Silva AC, Ogino S, Akarca FG, Freeman GJ, et al. Characterizing diversity in the tumor-immune microenvironment of distinct subclasses of gastroesophageal adenocarcinomas. Ann Oncol. 2020; 31:1011–20. 10.1016/j.annonc.2020.04.011 32387455 PMC7690253

[27] Huang W, Zhou K, Jiang Y, Chen C, Yuan Q, Han Z, Xie J, Yu S, Sun Z, Hu Y, Yu J, Liu H, Xiao R, et al. Radiomics Nomogram for Prediction of Peritoneal Metastasis in Patients With Gastric Cancer. Front Oncol. 2020; 10:1416. 10.3389/fonc.2020.01416 32974149 PMC7468436

[28] Sun KY, Hu HT, Chen SL, Ye JN, Li GH, Chen LD, Peng JJ, Feng ST, Yuan YJ, Hou X, Wu H, Li X, Wu TF, et al. CT-based radiomics scores predict response to neoadjuvant chemotherapy and survival in patients with gastric cancer. BMC Cancer. 2020; 20:468. 10.1186/s12885-020-06970-7 32450841 PMC7249312

[29] Wei XL, Xu JY, Wang DS, Chen DL, Ren C, Li JN, Wang F, Wang FH, Xu RH. Baseline lesion number as an efficacy predictive and independent prognostic factor and its joint utility with TMB for PD-1 inhibitor treatment in advanced gastric cancer. Ther Adv Med Oncol. 2021; 13:1758835921988996. 10.1177/1758835921988996 33613701 PMC7871293

[30] Wang F, Wei XL, Wang FH, Xu N, Shen L, Dai GH, Yuan XL, Chen Y, Yang SJ, Shi JH, Hu XC, Lin XY, Zhang QY, et al. Safety, efficacy and tumor mutational burden as a biomarker of overall survival benefit in chemo-refractory gastric cancer treated with toripalimab, a PD-1 antibody in phase Ib/II clinical trial NCT02915432. Ann Oncol. 2019; 30:1479–86. 10.1093/annonc/mdz197 31236579 PMC6771223

