# Supplementary material for: A radiomics model predicts the response of patients with advanced gastric cancer to PD-1 inhibitor treatment
Source: Aging (Albany NY). 2022 Jan 24;14(2):907–22. doi: 10.18632/aging.203850 (PMC8833127; doi:10.18632/aging.203850)
Supplement: Supplementary Text [file aging-14-203850-s001.pdf]

SUPPLEMENTARY TEXT

Radiomics score (Rad-score) calculation formula:

Logistic regression rad-score = -0.26449-1.02159*elongation–0.61826* log.sigma.0.5.mm.3D_firstorder_Kurtosis +0.58379* wavelet.HHL_firstorder_Mean

SVM rad-score = -0.04062-0.95159* elongation–0.56740* log.sigma.0.5.mm.3D_firstorder_Kurtosis +0.53031* wavelet.HHL_firstorder_Mean
