# Supplementary material for: A radiomics model predicts the response of patients with advanced gastric cancer to PD-1 inhibitor treatment
Source: Aging (Albany NY). 2022 Jan 24;14(2):907–22. doi: 10.18632/aging.203850 (PMC8833127; doi:10.18632/aging.203850)
Supplement: Supplementary Table 1 [file aging-14-203850-s002.pdf]

SUPPLEMENTARY TABLE

Supplementary Table 1. Performances of logistic regression and SVM classifiers.

Index	Training set (median, 95% CI)	Validation set (median, 95% CI)
Logistic Regression		
AUC	0.702 (0.694 - 0.711)	0.653 (0.623 - 0.684)
Sensitivity	0.694 (0.679 - 0.709)	0.659 (0.598 - 0.721)
Specificity	0.705 (0.687 - 0.724)	0.668 (0.613 - 0.724)
SVM		
AUC	0.695 (0.685 - 0.706)	0.636 (0.605 - 0.668)
Sensitivity	0.694 (0.674 - 0.715)	0.650 (0.587 - 0.714)
Specificity	0.690 (0.663 - 0.718)	0.650 (0.594 - 0.706)

Abbreviation: AUC, area under curve; CI, confidence interval; SVM: Support vector machines.
